# Microbial bioremediation products

**DOI:** 10.1111/1751-7915.12074

**Published:** 2013-08-14

**Authors:** Lawrence P Wackett

**Affiliations:** Department of Biochemistry, Molecular Biology & Biophysics, BioTechnology Institute, University of MinnesotaSt. Paul, MN, 55108, USA

**An annotated selection of world wide websites relevant to the topics in environmental microbiology**

## Evaluation of bioremediation products

### http://oil-clean.net/lib/images/sim.pdf

This is a peer-reviewed journal article discussing the efficacy of ten bioremediation products in the cleanup of oil that had been spilled along the Alaska coast.

## Bioremediation effectiveness

### http://ipec.utulsa.edu/Conf2002/prince_clark_lee_109.pdf

This paper describes the effectiveness of bioremediation for treating oil spilled in Alaska by the Exxon–Valdez tanker.

## DMOZ: Microbial remediation services

### http://www.dmoz.org/Science/Environment/Hazardous_Materials/Remediation_Technology/Biological_Remediation/Products_and_Services/

This site provides a large list of bioremediation products and services with links to the original websites describing the materials.

## Bioremediation: ASM library

### http://www.microbelibrary.org/library/resources/2776-bioremediation-and-treatment-of-industrial-waste

This site put up by the American Society for Microbiology provides a good summary of the principles and practices of bioremediation.

## Bioremediation: USGS

### http://water.usgs.gov/wid/html/bioremed.html

This site is dated but it contains specific examples of sites treated by bioremediation with links to further information.

## Microbes and their biodegradation pathways

### http://umbbd.ethz.ch/servlets/pageservlet?ptype=allmicros

This list is provided by the Biocatalysis/Biodegradation Database and has links for each organism to its known biodegradation pathways.

## CandidatusAccumulibacterphosphatis

### http://microbewiki.kenyon.edu/index.php/Candidatus_Accumulibacter_Phosphatis

*CandidatusAccumulibacterphosphatis* removes large amounts of phosphate from waters and stores it as polyphosphate; as such this microbe is useful in phosphate bioremediation and sequestration.

## CandidatusAccumulibacterphosphatisgenome project

### http://www.ncbi.nlm.nih.gov/genomeprj/29435

This webpage contains the genome sequence data for a phosphate-removing microbe used in phosphate remediation and sequestration.

## Bioremediation in the field search system: EPA

### http://www.epa.gov/ORD/dbases/bfss.html

This site provides information on waste sites at which bioremediation is being used or considered to be used.

## Hawaii Bioremediation Database

### http://www.hawaii.edu/abrp/dbase.html

This database defines some general terms and offers a brief list of bioremediation services provided within Hawaii.

## Frack water bioremediation

### http://futureenergy.ultralightstartups.com/campaign/detail/853

This site contains a description of a bioremediation technology for treating waters derived from unconventional oil and gas extraction, also known as hydraulic fracturing.

## Bioremediation of hydraulic fracturing wastewaters

### http://www.elsevierblogs.com/currentcomments/?p=814

This site contains an article that makes the case for using bioremediation as a tool for dealing with waters derived from hydraulic fracturing operations.

## Hydrocarbon treatment products: EPA page

### http://www.epa.gov/osweroe1/content/ncp/products/bioworld.htm

This is an example of a technical product bulletin for a bioremediation product posted by the U.S. Environmental Protection Agency.

## Regenesis products

### http://www.regenesis.com/contaminated-site-remediation-products/default.aspx

Regenesis' products for bioremediation generally revolve around the concept of reagent biostimulation to enhance the biodegradative activity of intrinsic microorganisms.

## JRW Bioremediation LLC

### http://www.jrwbioremediation.com

The product that is used by JRW Bioremediation is often determined by the conditions at the site and the decision of what substrate to add to support the appropriate bioremediation process.

## Bioremediation: Microbe Wiki

### http://microbewiki.kenyon.edu/index.php/Bioremediation

This page provides a general treatment of bioremediation and is found on the Microbe Wiki site.

## Bioremediation: When does it work?

### http://www.nap.edu/openbook.php?record_id=2131&page=16

This web book put out by the U.S. National Academy of Sciences is a general treatise on bioremediation.

## Molecular techniques in wastewater

### http://maciej.bioinfo.pl/pmid:16635533

This website provides describes microbial communities and methods of study that are involved in bioremediation to treat wastewater streams.

## United-Tech bioremediation

### http://www.united-tech.com/industries/industries/industries/bioremediation.html

This site describes the use of a microbial product to treat waste hydrocarbons from the oil and gas industries.

## CBI remediation

### http://www.cbi.com/markets/environmental/remediation-restoration

CBI is a large company that does many types of remediation and environmental restoration, including bioremediation.

## Haliburton bioremediation services

### http://www.halliburton.com/public/bar/contents/Data_Sheets/web/Sales_Data_Sheets/SDS-043.pdf

This subsidiary of Haliburton deals with the microbial remediation of hydrocarbons derived from oil and gas drilling.

## Microbes cleaning up Deepwater Horizon spill

### http://www.scientificamerican.com/article.cfm?id=how-microbes-clean-up-oil-spills

This article describes bioremediation, largely in the context of the Deepwater Horizon spill and the ability of microbes to handle oil and derivative wastes.

